# Changes in Spatiotemporal Parameters During Gait of Special Forces Operators with Additional External Load

**DOI:** 10.3390/s26061959

**Published:** 2026-03-20

**Authors:** Wojciech Paśko, Patryk Marszałek, Maciej Śliż, Krzysztof Maćkała, Cíntia França, Izabela Huzarska-Rynasiewicz, Rafał Podgórski, Élvio Rúbio Gouveia, Dominik Skiba, Krzysztof Przednowek

**Affiliations:** 1 Faculty of Physical Culture Sciences, Medical College of Rzeszów University, 35-959 Rzeszów, Poland; wopasko@ur.edu.pl (W.P.); msliz@ur.edu.pl (M.Ś.); ihuzarska@ur.edu.pl (I.H.-R.); krprzednowek@ur.edu.pl (K.P.); 2 Department of Track and Field, Wroclaw University of Health and Sport Sciences, 51-617 Wroclaw, Poland; krzysztof.mackala@awf.wroc.pl; 3 Department of Physical Education and Sport, University of Madeira, 9020-105 Funchal, Portugal; cintia.franca@staff.uma.pt (C.F.); erubiog@staff.uma.pt (É.R.G.); 4 LARSyS, Interactive Technologies Institute, 9020-105 Funchal, Portugal; 5 Department of Medicinal Chemistry and Metabolomics, Faculty of Medicine, Medical College of Rzeszów University, 35-959 Rzeszów, Poland; rpodgorski@ur.edu.pl; 6 Research Center in Sports Science, Health Sciences, and Human Development (CIDESD), 5000-801 Vila Real, Portugal; 7 CIPER—Interdisciplinary Center for the Study of Human Performance, Faculty of Human Kinetics, University of Lisbon, 1649-004 Lisbon, Portugal; 8 Physiotherapy Dominik Skiba, 39-120 Sędziszów Małopolski, Poland; dominiks989@gmail.com

**Keywords:** gait biomechanics, ground reaction force, pressure platform, load carriage, Special Forces Operators

## Abstract

Background: Gait with external load is an inherent element of military tasks, and the mass of equipment carried by soldiers has systematically increased over recent decades. Depending on the nature of the operation, soldiers may carry loads ranging from several to several dozen kilograms, which may affect gait biomechanics and increase the risk of overload injuries. The aim of this study was to evaluate changes in the spatiotemporal gait parameters of Special Forces Operators depending on the mass and type of the carried external load. Methods: The study included 34 active Special Forces Operators (age: 36.47 ± 5.63 years; height: 180.39 ± 5.72 cm; body mass: 85.92 ± 8.54 kg). Gait analysis was performed using an h/p/cosmos gaitway 3D + 1D treadmill equipped with an integrated pressure platform enabling ground reaction force (GRF) measurement. Participants performed gait trials at a speed of 5.5 km/h under four load conditions: 0 kg, 7 kg, 20 kg, and 27 kg. For each condition, 30 s measurement series were recorded, enabling analysis of a stable locomotion pattern and detection of gait phase events. Results: Statistically significant differences were demonstrated for the following parameters: stance phase, load response, single support, pre-swing, swing phase, double stance, foot rotation, step time, stride length, step width, cycle time, and cadence. The greatest changes were observed between unloaded gait and the condition with a helmet and vest. External load mainly caused prolongation of phases related to support and shortening of the swing phase and single support. Conclusions: Military load significantly modifies the temporal structure of gait in Special Forces Operators even at a constant, relatively low speed. The use of an instrumented treadmill with an integrated pressure platform and GRF measurement, as well as the registration of a large number of gait cycles, enabled the detection of subtle differences in spatiotemporal parameters and reliable assessment of stability and dynamic asymmetry under controlled laboratory conditions.

## 1. Introduction

Soldiers performing combat tasks are often additionally loaded with equipment, the mass of which depends on the nature of tactical operations [1,2]. Previous studies indicate that soldiers carry loads ranging from a few to several dozen kilograms [1,3,4,5,6,7]. Indian infantry soldiers carry loads ranging from 4.2 to 17 kg [8]. Experimental studies have examined the biomechanical effects of additional combat equipment weighing from several to approximately 21 kg [1,5]. Roy et al. [4] indicate that a ballistic vest alone may weigh up to 16 kg, and a backpack about 22 kg. In extreme cases, the mass of tactical equipment may be considerably higher. It has been reported that the total weight of full equipment may average 46.3 kg [6], while other studies have documented loads exceeding 40 kg among Swedish soldiers [7]. Marching with such loads may occur three to four times per week and last from several hours up to 17 h [2].

One consequence of prolonged gait with external load is changes in movement patterns that affect the spatiotemporal and kinematic parameters of gait [9,10]. In a study on German soldiers, a 15 kg backpack was found to increase skeletal muscle activity by an average of 75% [5]. The impact of external load can cause musculoskeletal injuries, and previous studies clearly indicate an increased risk of injury to the lower limbs and the spine [10,11,12,13]. High external load has also been observed to increase the risk of injury, which is confirmed by studies conducted on Australian soldiers [14].

Gait is the basic and natural form of human locomotion, ensuring autonomy and freedom of movement throughout life [15]. Contemporary research on gait analysis emphasizes the growing importance of identifying the most informative gait features and recognizing characteristic movement patterns, which enable the detection of even subtle differences between investigated conditions or groups [16]. The gait cycle constitutes a repetitive sequence of stance and swing phases, within which subphases corresponding to successive stages of body weight transfer and propulsion generation are distinguished. The described cycle is characterized by spatiotemporal parameters, which are among the key and most frequently analyzed variables in biomechanical gait assessment [17]. These parameters include, among others, step width and step length, cadence, step time, duration of individual phases, and gait speed [18,19]. An important indicator of gait pattern quality is also the asymmetry of spatiotemporal parameters between the right and left limbs, which may lead to increased energy cost and be associated with deficits in dynamic balance [20].

Previous studies in the field of soldier gait biomechanics have come to divergent conclusions regarding kinematic and spatiotemporal adaptations, which may result from the heterogeneity of applied research methods, experimental protocols, and measurement tools [10]. Analysis of the available studies suggests that spatiotemporal gait adaptations may show lower sensitivity compared to kinematic changes, and the observed differences often do not exceed a few percent, which may remain beyond the detection range of many measurement instruments [11,13].

In the assessment of small differences in the spatiotemporal structure of gait, the registration of many consecutive gait cycles under repeatable conditions is of key importance, as it enables stabilization of the analyzed locomotion pattern and reliable detection of subtle adaptive changes [21]. Precise detection of events such as heel contact with the ground or toe-off, which define individual phases of the gait cycle, also plays an important role [22,23,24]. In the context of the assessment of these events, ground reaction forces (GRFs), which reflect the actual load acting on the human locomotor system, are of key importance [25,26,27]. Previous studies also show that even a small load of 3.5 kg carried by uniformed service officers may significantly increase the level of GRF asymmetry between limbs [28]. Due to the subtle nature of kinematic and kinetic gait adaptations in soldiers under load, reliable assessment of changes in the spatiotemporal structure and in the course of GRFs requires the use of measurement systems with high resolution, sensitivity, and repeatability [10,29,30].

Despite the existence of studies on changes in spatiotemporal gait parameters under the influence of load, there is a lack of studies in which a wide range of these parameters are analyzed simultaneously using tools capable of detecting even small changes.

Special Forces Operators represent an elite and highly selected subgroup within military units, and their level of physical fitness is often compared to that of elite athletes [31]. Moreover, previous studies indicate that Special Forces Operators may exhibit higher cardiorespiratory fitness compared with conventional military soldiers [32], which may potentially represent one of the factors influencing gait adaptation strategies. Moreover, the energy requirements of Special Forces soldiers are also higher than those of an average soldier, which is associated with their greater level of physical activity [33].

Consequently, the aim of the present study was to analyze changes in spatiotemporal gait parameters among Special Forces soldiers while moving with additional load. The analysis was conducted under four conditions: in sports clothing, in a ballistic vest and helmet, with a backpack weighing 20 kg, and in a ballistic vest and helmet with a backpack. Additionally, asymmetry of spatiotemporal parameters and ground reaction forces during gait in individual load conditions was performed.

## 2. Materials and Methods

### 2.1. Materials

Thirty-four Polish Special Forces Operators aged 36.47±5.63 years participated in the study. The soldiers were characterized by a body height of 180.39±5.72 cm, a body mass of 85.92±8.54 kg and a BMI of 26.35±1.67$\frac{\mathrm{kg}}{m^{2}}$. The subjects’ body height was measured using a stadiometer (Seca stadiometer, Hamburg, Germany), while body mass was measured using a Tanita DC-360 (Tanita Corporation, Tokyo, Japan). All operators had completed the selection stage and training to become a soldier of a special unit, which, in total, lasted approximately 4 years. The respondents were active operators of a special unit who belonged to the Water or Air Force Division, and their seniority ranged from 5 to 8 years. Only participants who were fully rested and without any health complaints took part in the study. The participants voluntarily participated in the study and gave written consent.

The study scope and design were approved by the Ethics Committee of the University of Rzeszów/Poland (resolution 2023/12/0060 of 6 December 2023). All procedures performed in studies involving human participants were in accordance with the ethical standards of the Ethics Committee of the University of Rzeszów and with the 1964 Helsinki Declaration and its later amendments.

### 2.2. Methods

#### 2.2.1. Study Design

Gait analysis was carried out in four conditions. The first measurement (0 kg of external load) consisted of a gait analysis only in sports attire. During the second measurement (7 kg of external load), the subjects additionally wore a helmet and a bulletproof vest. The helmet weighed 1 kg and the bulletproof vest weighed 6 kg, for a total of 7 kg. The third measurement (20 kg external load) consisted of walking in sports attire with a military backpack, which weighed 20 kg. The final measurement (27 kg external load) consisted of walking with a bulletproof vest, helmet and backpack, with a total load of 27 kg. During each measurement, the subjects wore the same footwear and sports attire. The measurements were conducted at an imposed gait speed of 5.5 km/h and a treadmill elevation of 0 degrees. The speed was not adjusted for body height, as in a military setting, soldiers have to march at a constant speed, regardless of their anthropometry [34]. A speed of 5.5 km/h was chosen because it is between the average walking speed of most healthy adults (5 km/h) [35] and the typical military walking speed (6 km/h) [34]. In addition, during the selection process, candidates must complete a special marathon with load within a certain time, and the speed at which they should move to complete this stage of the selection is about 5.5 km/h. Soldiers performed one trial for each of the listed test conditions. The trial was repeated if there was a gait disturbance due to tripping or loss of balance. Data were recorded for 30 s for each trial. Data recording started when the treadmill accelerated to a given speed and the subject had adapted to the measurement conditions. A 3 min rest period between measurements was used to prevent fatigue. The tests were conducted at 21.8 °C, 1004.25 hPa and 57.4% humidity.

#### 2.2.2. Measuring Tools

**Figure 1:**
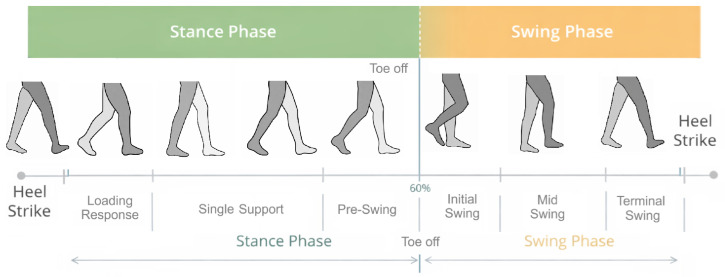
Phases of gait. Source: Authors’ elaboration based on Noraxon (https://www.noraxon.com/gait-analysis-running-analysis/, accessed on 20 December 2025).

The analysis of the soldiers’ gait was carried out with the h/p/cosmos gaitway 3D + 1D treadmill, which operates in the speed range from 0 to 40 km/h and elevation range from −20% to +20%. The size of the transmission belt is 190 × 65 cm and the size of the measurement matrix is 155 × 54.1 cm. The platform contains 11,264 sensors, integrated into the treadmill structure, which allow 1D and 3D force measurements. The dynamic measurement module allows the analysis of the distribution of foot pressure forces, the spatiotemporal parameters of gait and the analysis of individual gait phases. For the analysis of gait parameters, the software Noraxon MR3 (version 3.18.10, Noraxon USA Inc., Scottsdale, AZ, USA) and Gaitway 3D (version 1.7.7, RTE 14.0-H/P/Cosmos Sports & Medical GmbH and Arsalis SRL, Glabais, Belgium) were used. The data sampling rate for the platform was 100 Hz. The Noraxon system was responsible for data processing and signal filtering. Three main groups of spatiotemporal parameters were identified: gait phase parameters, gait spatial parameters and gait time parameters. The first group of parameters presented in Figure 1 describes the percentage composition of the different gait phases, such as stance phase, load response, single support, pre-swing, swing phase, and double stance. Gait events were identified based on vertical ground reaction force (Fz) threshold crossings (contact defined as Fz > 50 N), and phase boundaries were determined from the sequence of these contact events and the left/right force distribution.

The spatial gait parameters include foot rotation (deg), step length (cm), stride length (cm) and step width (cm). Gait time parameters are the last group of spatiotemporal parameters, comprising: step time (ms), stride time (ms) and cadence (steps/min). The first two parameters represent the duration of the walking step and one whole gait cycle, respectively. Cadence indicates the number of steps taken per minute.

### 2.3. Statistical Method

Statistical analysis was performed in Jamovi ver. 2.4.8. The first step was to verify the normal distribution for all spatiotemporal parameters using the Shapiro–Wilk test. Each variable showed a normal distribution (p>0.05); therefore, the arithmetic mean and standard deviation were used to characterize all variables. Additionally, the assumption of homogeneity of variances was verified using the Levene test. The analysis took into account that all analyzed variables assumed homogeneity of variances (p>0.05). The statistical significance of differences between measurements was determined using an ANOVA test for repeated measures. In addition, the effect size was calculated using $\eta^{2}$. The next step was a post hoc analysis performed using the Bonferroni test to compare changes in spatiotemporal parameters across conditions. In addition, the Benjamini–Hochberg procedure was applied to control the false discovery rate using R (v4.3.3, Vienna, Austria) and RStudio (v2023.12.1+402, Boston, MA, USA). Evaluation of the symmetry index was carried out using the Robinson index [36]:

$$SI=\frac{2\cdot (\mathrm{higher}\;\mathrm{value}-\mathrm{lower}\;\mathrm{value})}{\mathrm{total}\;\mathrm{value}}\cdot 100\%$$

The symmetry indices did not show a normal distribution; therefore, the values are presented using the median and interquartile range (IQR). The statistical significance of the differences between the symmetry index for the spatiotemporal parameters was calculated using the Friedman test. The magnitude of the effect was demonstrated using Kendall’s concordance coefficient. In the case of statistically significant differences, a post hoc analysis was performed using the Durbin–Conover test.

**Figure 2:**
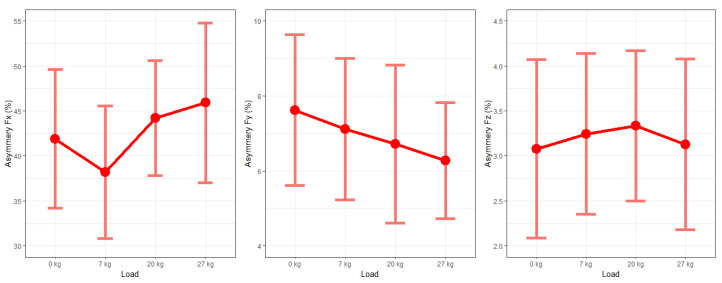
Asymmetry index for ground reaction forces in three axes: Fx, Fy, Fz.

The symmetry for ground reaction forces was also represented by the Robinson index [36]. The analysis of the symmetry of the ground reaction force is presented by means of a line graph, showing the arithmetic mean and 95% confidence intervals for each loading condition. Analysis of statistically significant differences between loads was performed using the Friedman test. Line graphs of ground reaction forces (Figure 2) were created using R 4.3.1 software.

## 3. Results

Table 1 shows a comparison of spatiotemporal parameters across the four gait conditions. The analysis showed statistically significant differences in stance phase, load response, single support, pre-swing, swing phase, foot rotation and step time for both the left and right lower limbs. Data for the left and right limbs are presented separately in order to provide precise values of limb-specific responses to increasing external load. For stance phase, load response and pre-swing, the highest values were recorded during gait analysis under full-load conditions (27 kg of external load), while the lowest values were recorded during gait in sportswear only (0 kg of external load). For single support and swing phase, the highest values were recorded during gait in sportswear only, and the lowest values were observed during full load. The highest values for the left-foot rotation were recorded during gait with a bulletproof vest and helmet (7.57±3.64 deg), while the lowest values were recorded during gait with a backpack only (6.15±3.89 deg). Similarly, for the right-foot rotation the highest values were obtained for gait with a bulletproof vest and helmet (9.08±4.05 deg), but the lowest values were observed for gait with full load (7.44±3.97 deg). Step time for both the left and right lower limbs showed the highest values during gait in sportswear alone, while the lowest values were recorded during gait with a bulletproof vest and helmet.

In addition, statistically significant differences were observed for parameters such as double stance, stride length, step width, stride time and cadence. The highest values for double stance were observed for gait with full load, and the lowest values during gait without additional load. For stride length, similar values were found during gait in athletic attire alone and with full load (156.79±4.70 cm and 156.28±4.55 cm, respectively). Similar results for stride length were also obtained for walking with a bulletproof vest and helmet (155.68±5.05 cm) and walking with a backpack alone (155.98±4.78 cm). Step width reached the highest value during walking with full load, while the lowest value of this parameter was recorded during walking without a load. Stride time had the highest value during gait in athletic attire alone (1032.01±31.38 ms), while the lowest result was recorded during gait with a bulletproof vest and helmet (1024.14±33.23 ms). The highest cadence was observed during gait with a bulletproof vest and helmet (117.31±3.81 step/min), while a fairly similar value was also recorded during gait with a backpack alone (117.00±3.60 step/min). The lowest cadence was found when walking in sportswear alone (116.40±3.50 step/min), but a similar value was also recorded when walking under full load at 116.75±3.35 step/min.

For spatiotemporal parameters such as step length for both the left and right lower limbs, no significant statistical differences were found.

The analysis of the symmetry index is shown in Table 2. It should be noted that the closer the value is to zero, the more symmetrical the gait. The highest values of gait asymmetry were observed for foot rotation. The highest asymmetry was observed when walking with a load of 20 kg and was 49.39%. The lowest asymmetry values were observed during gait with a 27 kg load for the stance phase. Statistically significant differences were found only for pre-swing asymmetry. The highest asymmetry rate was observed when walking without a load, while the lowest asymmetry rate was observed during walking with a 20 kg load.

Statistically significant differences in spatiotemporal parameters were found in a post hoc analysis, presented in Table 3. Statistically significant differences were detected between gait in sportswear alone and gait in a bulletproof vest and helmet for every parameter except foot rotation (left and right lower limb) and step width. For the differences between gait in sportswear alone and gait with a backpack, no statistically significant differences were observed for stride length, step time (left and right lower limbs), stride time or cadence. When comparing walking in sportswear and walking at full load, the analysis showed no significant differences in stride length, step time (left and right lower limbs), stride time and cadence.

Comparison between gait with a bulletproof vest and helmet and gait with a backpack showed no significant changes in parameters such as stride length, step width, step time (left and right lower limbs), stride time and cadence. Comparison of gait with a bulletproof vest and helmet with gait under full load showed no statistically significant differences in stride length, step time (left and right lower limbs), stride time and cadence. Statistically significant differences between gait with a backpack and gait under full load were observed only for stance phase (right lower limb), load response (left and right lower limbs), single support (left lower limb), pre-swing (left and right lower limbs), swing phase (right lower limb) and double stance.

Figure 2 shows the asymmetry of maximum ground reaction forces recorded during the 30 s test trial. The force changes are measured in three planes and four external load conditions. It is worth noting that the highest values of the symmetry index were observed for the x-axis force, while the lowest values were observed for the z-axis force. In the case of Fx, the lowest asymmetry was observed during gait with a 7 kg load. The highest value of the symmetry index was obtained during a load of 27 kg. For Fy, a gradual decrease in asymmetry was observed as the load increased. For Fz, a gradual increase in asymmetry was observed up to a load of 20 kg. When the load was increased to 27 kg, there was a decrease in the symmetry index. There were no statistically significant differences between the symmetry index and the additional load in each of the three axes.

## 4. Discussion

The aim of the present study was to evaluate changes in the spatiotemporal gait parameters of Special Forces Operators depending on the mass and type of carried external load. Statistically significant differences were identified in the analyzed spatiotemporal parameters in response to additional external load. The use of a treadmill with a built-in pressure platform enabled the registration of consecutive gait cycles under conditions of high repeatability and precise determination of individual phases and subphases of the gait cycle.

The present findings are partially consistent with previous reports, particularly with respect to changes in the temporal structure of the gait cycle associated with increasing load [9,29,37,38,39]. The observed changes in the stance phase were also reported by Attwells et al. [37], who applied four load variants in their study and found prolongation of the stance phase in each of them. Comparison of spatiotemporal parameters between individual gait conditions showed significant changes in the load response phase, single support, pre-swing phase, swing phase, and double stance, with the strongest differences observed between the unloaded and light-load conditions. Similar results were obtained by Sousa et al. [38], who observed changes in the swing phase and in the double stance phase with increasing load. The importance of changes in the double stance phase was emphasized by Fellin et al. [29] and Bode [39], suggesting that it may represent one of the more sensitive parameters to external loading. Dar et al. [9] demonstrated significant prolongation of the double stance phase after adding external load. In contrast, Majumdar et al. [8] did not note significant changes in single support, swing phase, or stance phase in soldiers with increasing loads. However, the study by Majumdar et al. [8] included as many as nine load variants with relatively small mass differences. This could have been the reason for the lack of observed differences in spatiotemporal parameters. Moreover, the use of the optoelectronic HiRes Expert Vision System (Motion Analysis Corporation) by Majumdar et al. [8], in which gait phases were determined indirectly from marker kinematics, could have made it difficult to detect small changes in parameters such as double stance, load response, or pre-swing. In contrast, the pressure platform with GRF measurement used in the present study allowed direct detection of phase events, which may have increased the accuracy of spatiotemporal parameter assessment.

In the conducted study, a decrease in foot rotation was observed when the load difference was at least 13 kg. In the case of step width, an increase in this parameter was demonstrated with a load difference of 20 kg. This means that foot rotation and step width may change in value with larger differences in the mass of the carried load. However, in a study conducted among police officers, it was shown that a small load of 3.5 kg caused a significant increase in the foot rotation parameter [40]. In the study by Kasović et al., a pressure platform 1.5 m long within a 10.5 m walkway was used, and the recording included only five trials in each load configuration. This means a smaller number of stable gait cycles, which could limit the sensitivity to detect subtle changes in spatiotemporal parameters. Although a significant increase in foot rotation was demonstrated, the effect size was small, which, similarly to the present study, indicates a limited scale of the observed changes. Analysis of the study by Sessoms et al. [41] did not show significant differences in step width depending on the additional load, which amounted to 18 kg, 25 kg, or 34 kg. In their study, load distribution systems were used, which could stabilize the trunk position and limit compensatory increases in step width.

In the present study, statistically significant differences in stride length, step time, stride time, and cadence were observed only between gait in sports clothing alone and gait in a ballistic vest and helmet. Adding a small external load resulted in a decrease in stride length, step time, and stride time, with a simultaneous increase in cadence. Similar results for stride length and cadence were presented by Fellin et al. [29] and Bode et al. [39]. Attwells et al. [37] also showed the greatest differences in gait parameters after adding a light load (8 kg). However, in their study an increase in stride length was observed after increasing the load by 8 kg compared to control conditions [37]. This discrepancy may result from differences in experimental conditions. In the study by Attwells et al. [37], soldiers performed trials at a self-selected speed, which allowed them to freely manipulate cadence and step length according to individual adaptive strategies. Nevertheless, both in the present study and in the work of Attwells et al. [37], a common feature of the low-load conditions was the presence of a helmet. This may suggest that not only the magnitude of the load but also its type, placement, and mode of carriage may play a key role in shaping the observed adaptive gait mechanisms. Stride length under load was also analyzed by Krupenevich et al. [42]. With a load of 22 kg, a 1.3% shortening of stride length was demonstrated compared to unloaded gait [42]. Dar et al. [9] did not observe significant changes in stride time or cadence, although it should be noted that their study used a load equal to 50% of body mass. Moreover, a statistically significant reduction in stride length after adding the load was demonstrated [9]. The lack of changes in temporal parameters in the study by Dar et al. [9] may suggest that at very high relative loads, the ability to further modulate cadence and stride time is limited, and biomechanical adaptations occur mainly at the level of body posture and joint kinematics rather than basic spatiotemporal parameters.

Kasović et al. [43] did not show statistically significant differences in the analyzed spatiotemporal parameters (including step length, stride length, stride time, cadence), although significant changes in kinetic variables were observed with increasing load. The lack of significance of spatiotemporal parameters may result from the use of overground walking, which is associated with recording a limited number of stable gait cycles and potentially lower sensitivity of the analysis to subtle adaptations. Similarly, Majumdar et al. [8] did not demonstrate significant changes in step length, stride length, or cadence, despite observed trends. In their study, overground walking on a 10 m section and an optoelectronic motion analysis system were used, in which the phases and subphases of the gait cycle were determined indirectly based on marker kinematics rather than directly from ground reaction forces. Such a protocol could limit the ability to detect small changes in the temporal structure of the gait cycle. The results of the studies by Majumdar et al. [8] and Kasović et al. [43] suggest that adaptations to external load may be relatively small at the level of basic spatiotemporal parameters, while being more clearly manifested in kinetic variables and locomotor system kinematics.

The results of previous studies remain inconclusive [10]. A literature review [10] showed that among eight studies conducted with military personnel, five did not find a statistically significant relationship between the magnitude of the carried load and spatiotemporal gait parameters [10]. On the one hand, some previous works confirm the results obtained in the present study [29,37,39], while on the other hand, some authors did not demonstrate changes in the analyzed parameters with increasing external load [8,43].

One of the reasons for these discrepancies may be the diversity of measurement methods and research tools. Differences in the values of spatiotemporal parameters, regardless of the carried load, are usually small. Therefore, the ability to detect them largely depends on the sensitivity and accuracy of the applied measurement system. Consequently, the choice of an appropriate method seems crucial for reliable assessment of the variability of gait parameters under the influence of external load.

Among the analyzed symmetry indices, only pre-swing asymmetry showed statistically significant differences across load conditions. The results suggest that adding external load may cause a reduction in pre-swing phase asymmetry between the left and right lower limbs; however, no significant changes in asymmetry were observed between individual load levels. Similarly, for the remaining spatiotemporal parameters, no significant differences were found between different load conditions. A similar analysis was conducted in police recruits, where after adding a load of 3.5 kg, a significant change in pre-swing asymmetry was noted, but it was an increase [44]. These authors also observed significant changes in parameters such as stance phase, load response, single support, swing phase, and step time [44]. The present results also indicate that asymmetry of ground reaction forces does not change significantly with additional load, which may result from an even distribution of force increase between the lower limbs. Different conclusions were presented by Kasović et al. [45], who observed an increase in GRF asymmetry after adding external load. It is suggested, however, that postural adaptation and force distribution depend on the type of carried load and professional experience [20], and moreover, load placed close to the body center of mass, such as a backpack, causes the least gait disturbance [46].

One limitation of the present study was the constant walking speed imposed by the treadmill, without normalization to body height or lower limb length and without the use of individualized walking speeds. Therefore, the obtained results should be interpreted as gait adaptations under standardized marching conditions rather than fully individualized locomotor strategies. However, this approach allowed for control of gait variability and reflects military conditions, in which soldiers are required to adjust their marching pace to the group. Additional limitations included the absence of a weapon and a full combat uniform as components of the carried load, which may have influenced the analyzed gait parameters. The distribution of individual load components is also a relevant factor, as it may affect the magnitude and character of the observed gait adaptations. Moreover, the use of athletic footwear may have altered gait patterns compared to tactical footwear.

Another limitation was the lack of kinematic analysis, which prevented the assessment of adaptations in individual lower limb joint angles and limited the ability to directly relate changes in spatiotemporal parameters to kinematic adaptations. Furthermore, the applied experimental protocol was limited to short-term measurements and did not allow for evaluation of the cumulative effects of prolonged loaded marching or adaptations related to fatigue. Differences resulting from the use of treadmill walking compared with overground walking should also be considered. Although laboratory-based measurements provide high control over experimental conditions, they may not fully reflect natural locomotor patterns, which could be more comprehensively assessed under field conditions.

An important strength of the conducted research was the use of a treadmill with a pressure platform, which enabled the registration of several dozen gait cycles and precise measurement of 13 parameters. The use of measurements based on direct registration of ground reaction forces enabled accurate detection of gait phase events, which increases the sensitivity of the analysis compared to systems based solely on marker kinematics. Analysis of additional parameters allows for a better assessment of the impact of load on soldier gait. Previous studies on the influence of load on soldier gait focused mainly on basic spatiotemporal parameters such as step length, cadence, stance time, double support time, or foot rotation. The results of the present study indicate that most changes occur primarily in the duration of individual gait phases, which have received little attention so far.

The presented research provides detailed information on the spatiotemporal gait parameters of soldiers. Such analyses are important because they deepen knowledge about the kinematic and energetic aspects of gait in this specific population. As in previous works, the magnitude of the observed changes is relatively small, but they may be significant, especially during prolonged gait with increasing load. Knowledge about the impact of external load enables better prediction and implementation of preventive strategies in the field of overload injuries. The obtained results suggest that the use of a treadmill with a pressure matrix and GRF measurement, with registration of a large number of gait cycles, enables the detection of small differences in spatiotemporal parameters. The conducted research also indicates the need to seek a compromise between the amount of necessary equipment carried by soldiers and the risk of injuries and unfavorable changes in gait kinematics.

Future research may focus on comparing spatiotemporal parameters between Special Forces soldiers and the broader military population or a control group. Moreover, it is possible to conduct observations of changes in spatiotemporal parameters during prolonged gait with load in field conditions, with full tactical equipment. It is also worth considering combining laboratory measurements using pressure platforms with inertial sensor systems, as well as taking into account the influence of changes in marching speed, which may significantly affect the analyzed parameters.

## 5. Conclusions

Military load in the form of a backpack, tactical vest, and helmet significantly affects gait biomechanics of SFO operators. The present study demonstrated changes in spatiotemporal parameters under four load conditions, with relatively constant stride length, which may indicate an adaptive locomotion strategy.

Loads in the range of 7–27 kg resulted in modifications of temporal gait parameters, leading to prolongation of the stance and double support phases and shortening of the single support and swing phases. Such changes, accumulating during prolonged marches, may increase the risk of overload injuries, and the obtained results may support the monitoring of training loads and early identification of risk factors in soldiers performing tasks with additional equipment.

## Figures and Tables

**Table 1:** Numeral characteristics of gait in four load conditions.

Variable	0 kg	7 kg	20 kg	27 kg	p	e.s.
	$\bar{x}\;\pm \;\mathrm{sd}$	$\bar{x}\;\pm \;\mathrm{sd}$	$\bar{x}\;\pm \;\mathrm{sd}$	$\bar{x}\;\pm \;\mathrm{sd}$		$\eta^{2}$
Stance Phase [%]—l	63.11±0.89	63.50±0.86	64.48±0.85	64.72±0.85	<0.001 *	0.38
Stance Phase [%]—r	63.12±0.95	63.55±0.95	64.45±0.89	64.76±0.87	<0.001 *	0.35
Load Response [%]—l	13.14±0.93	13.60±0.89	14.57±0.90	14.88±0.89	<0.001 *	0.39
Load Response [%]—r	13.10±0.87	13.47±0.86	14.36±0.81	14.60±0.81	<0.001 *	0.36
Single Support [%]—l	36.88±0.94	36.45±0.95	35.54±0.88	35.23±0.88	<0.001 *	0.35
Single Support [%]—r	36.88±0.89	36.49±0.85	35.53±0.86	35.29±0.85	<0.001 *	0.38
Pre-swing [%]—l	13.11±0.86	13.48±0.85	14.36±0.81	14.60±0.82	<0.001 *	0.36
Pre-swing [%]—r	13.14±0.93	13.59±0.89	14.57±0.90	14.89±0.89	<0.001 *	0.39
Swing Phase [%]—l	36.89±0.89	36.50±0.86	35.52±0.85	35.28±0.85	<0.001 *	0.38
Swing Phase [%]—r	36.88±0.95	36.45±0.95	35.55±0.89	35.24±0.87	<0.001 *	0.35
Double Stance [%]	26.24±1.66	27.07±1.65	28.93±1.57	29.48±1.59	<0.001 *	0.41
Foot Rotation [deg]—l	7.33±3.80	7.57±3.64	6.15±3.89	6.21±4.02	<0.001 *	0.03
Foot Rotation [deg]—r	8.70±3.89	9.08±4.05	7.61±3.80	7.44±3.97	<0.001 *	0.03
Step Length [cm]—l	78.83±2.48	78.27±2.68	78.55±2.41	78.72±2.49	0.059	0.01
Step Length [cm]—r	77.95±2.62	77.40±2.68	77.43±2.64	77.55±2.36	0.060	0.01
Stride Length [cm]	156.79±4.70	155.68±5.05	155.98±4.78	156.28±4.55	0.039 *	0.01
Step Width [cm]	11.17±3.01	11.33±2.96	11.74±2.85	12.12±3.07	<0.001 *	0.02
Step Time [ms]—l	515.91±16.52	511.76±17.69	512.15±16.23	513.16±15.35	0.026 *	0.01
Step Time [ms]—r	516.10±16.41	512.38±16.79	514.54±16.61	515.66±15.58	0.038 *	0.01
Stride Time [ms]	1032.01±31.38	1024.14±33.23	1026.69±31.59	1028.83±29.7	0.024 *	0.01
Cadence [step/min]	116.40±3.50	117.31±3.81	117.00±3.60	116.75±3.35	0.018 *	0.01

l—left; r—right; $\bar{x}$—mean value; sd—standard deviation; p—probability of testing; e.s.—effect size; *—statistical significance.

**Table 2:** Numerical characteristics of gait symmetry under four loadings.

Symmetry [%]	0 kg	7 kg	20 kg	27 kg	p	e.s.
	ME±IQR	ME±IQR	ME±IQR	ME±IQR		***W***
Stance Phase	0.78 ± 0.81	0.97 ± 0.87	0.93 ± 0.95	0.84 ± 0.64	0.638	0.02
Load Response	3.57 ± 3.51	3.29 ± 3.46	2.62 ± 3.46	2.67 ± 3.78	0.103	0.06
Swing Phase	1.32 ± 1.34	1.79 ± 1.53	1.72 ± 1.66	1.51 ± 1.18	0.196	0.05
Step Length	1.67 ± 1.84	1.47 ± 2.00	1.43 ± 2.31	1.29 ± 2.29	0.024 *	0.09
Foot Rotation	43.91 ± 43.79	44.98 ± 38.16	47.77 ± 55.00	49.39 ± 62.92	0.35	0.03
Step Time	1.00 ± 1.95	1.11 ± 1.70	1.11 ± 1.67	1.30 ± 1.38	0.392	0.03
Single Support	1.40 ± 1.22	1.80 ± 1.52	1.75 ± 1.59	1.51 ± 1.11	0.563	0.02
Pre-swing	3.49 ± 3.67	3.35 ± 3.68	2.73 ± 3.69	2.80 ± 4.05	0.527	0.02

ME—median; IQR—interquartile range; p—probability of testing; e.s.—effect size; *—statistical significance; W—Kendall’s coefficient of concordance.

**Table 3:** Post hoc analysis between different loads.

Variable	0 kg	0 kg	0 kg	7 kg	7 kg	20 kg
	**vs.**	**vs.**	**vs.**	**vs.**	**vs.**	**vs.**
	**7 kg**	**20 kg**	**27 kg**	**20 kg**	**27 kg**	**27 kg**
Stance Phase [%]—l	***	***	***	***	***	NS
Stance Phase [%]—r	***	***	***	***	***	***
Load Response [%]—l	***	***	***	***	***	**
Load Response [%]—r	***	***	***	***	***	*
Single Support [%]—l	***	***	***	***	***	***
Single Support [%]—r	***	***	***	***	***	NS
Pre-swing [%]—l	***	***	***	***	***	*
Pre-swing [%]—r	***	***	***	***	***	**
Swing Phase [%]—l	***	***	***	***	***	NS
Swing Phase [%]—r	***	***	***	***	***	***
Double Stance [%]	***	***	***	***	***	**
Foot Rotation [deg]—l	NS	***	**	***	***	NS
Foot Rotation [deg]—r	NS	***	***	***	***	NS
Stride Length [cm]	**	NS	NS	NS	NS	NS
Step Width [cm]	NS	*	**	NS	*	NS
Step Time [ms]—l	**	NS	NS	NS	NS	NS
Step Time [ms]—r	*	NS	NS	NS	NS	NS
Stride Time [ms]	**	NS	NS	NS	NS	NS
Cadence [step/min]	**	NS	NS	NS	NS	NS
Pre-swing Asymmetry [%]	**	*	*	NS	NS	NS

l—left; r—right; NS—no statistical significance; *—p<0.05; **—p<0.01; ***—p<0.001.

## Data Availability

The data presented in this study are available on request from the corresponding author.

## DURC Statement

Current research is limited to the field of biomechanics and gait analysis, which is beneficial for understanding human movement and improving performance and injury prevention and does not pose a threat to public health or national security. Authors acknowledge the dual use potential of the research involving biomechanical data analysis and gait assessment methodologies and confirm that all necessary precautions have been taken to prevent potential misuse. As an ethical responsibility, authors strictly adhere to relevant national and international laws about DURC. Authors advocate for responsible deployment, ethical considerations, regulatory compliance, and transparent reporting to mitigate misuse risks and foster beneficial outcomes.
